# Telerehabilitation for Word Retrieval Deficits in Bilinguals With Aphasia: Effectiveness and Reliability as Compared to In-person Language Therapy

**DOI:** 10.3389/fneur.2021.589330

**Published:** 2021-05-20

**Authors:** Claudia Peñaloza, Michael Scimeca, Angelica Gaona, Erin Carpenter, Nishaat Mukadam, Teresa Gray, Shilpa Shamapant, Swathi Kiran

**Affiliations:** ^1^Aphasia Research Laboratory, Department of Speech, Language and Hearing Sciences, Boston University, Boston, MA, United States; ^2^Gray Matter Laboratory, Department of Speech, Language and Hearing Sciences, San Francisco State University, San Francisco, CA, United States; ^3^Austin Speech Labs, Austin, TX, United States

**Keywords:** bilingual aphasia, telerehabilitation, videoconference, language therapy, semantic feature analysis, reliability, treatment fidelity, treatment effectiveness

## Abstract

**Background:** Bilinguals with post-stroke aphasia (BWA) require treatment options that are sensitive to their particular bilingual background and deficits across languages. However, they may experience limited access to bilingual clinical resources due to reduced availability of bilingual practitioners, geographical constraints, and other difficulties. Telerehabilitation can improve access to bilingual clinical services for BWA and facilitate the delivery of specific language treatments at distance, but more evidence on its effectiveness and reliability is needed. This study aimed to determine the equivalence of effectiveness and reliability of a semantic treatment for word retrieval deficits in BWA delivered via telerehabilitation relative to in-person therapy.

**Methods:** We examined the retrospective data of 16 BWA who received 20 sessions of therapy based on semantic feature analysis for word retrieval deficits in person (*n* = 8) or via telerehabilitation (*n* = 8). The two groups were comparable on age, years of education, time of post-stroke onset, aphasia severity, and naming ability in both languages. Treatment effectiveness (i.e., effect sizes in the treated and the untreated language, and change on secondary outcome measures) and reliability (i.e., clinician adherence to treatment protocol) were computed for each delivery modality and compared across groups.

**Results:** Significant improvements were observed in most patients, with no significant differences in treatment effect sizes or secondary outcomes in the treated and the untreated language between the teletherapy group and the in-person therapy group. Also, the average percentage of correctly delivered treatment steps by clinicians was high for both therapy delivery methods with no significant differences between the telerehabilitation vs. the in-person modality.

**Discussion:** This study provides evidence of the equivalence of treatment gains between teletherapy and in-person therapy in BWA and the high reliability with which treatment for word retrieval deficits can be delivered via telerehabilitation, suggesting that the essential treatment components of the intervention can be conducted in a comparable manner in both delivery modalities. We further discuss the benefits and potential challenges of the implementation of telerehabilitation for BWA. In the future, telerehabilitation may increase access to therapy for BWA with varying linguistic and cultural backgrounds, thus, offering a more inclusive treatment approach to this population.

## Introduction

Over the last few decades, telerehabilitation has motivated a growing interest across different fields of rehabilitation practice due to its potential to improve accessibility to clinical services for individuals with varying assessment, intervention, and consultation needs. Telerehabilitation entails the use of telecommunications and information technology to provide rehabilitation services at distance (1) and includes videoconference, patient portals or platforms, virtual reality, mobile applications, wearable and therapeutic gaming technologies, and other Internet-based methods that facilitate clinician–patient interactions (2). Telerehabilitation (or telepractice as endorsed by the American Speech–Language–Hearing Association, ASHA, 2020) is particularly relevant to the field of speech and language therapy given its great potential to deliver clinical services via videoconference and through interactive computer-based therapy activities (3) providing a highly suitable method to facilitate language-focused interventions that typically rely on audiovisual interactions (4). Importantly, a recent systematic review of telepractice for adult speech and language pathology services supports its use as an appropriate clinical service delivery model for different adult populations including people with post-stroke aphasia (5) who often experience long-lasting and chronic disability. However, this work also highlights the lack of research examining the benefits and limitations of using telerehabilitation with bilingual adults with post-stroke aphasia (BWA), a clinical population that requires access to bilingual clinicians who can provide high-quality assessment and treatment options that are sensitive to the particular linguistic characteristics and needs that make them different from their monolingual counterparts. To fill this gap in the literature, the present study aims to examine treatment effectiveness and reliability in a semantic-based intervention for word retrieval deficits in BWA delivered via telerehabilitation compared with in-person therapy.

As societies become more multicultural and multilingual, the higher incidence of stroke in older individuals from diverse racial, ethnic, and linguistic backgrounds is expected to result in an increased presence of bilingual adults in neurorehabilitation programs (6). Aphasia is the most common speech and language disorder encountered in bilingual adults after a stroke leading to deficits that may differ across their two languages in terms of the specific language domains affected and their overall severity (7, 8). Word retrieval deficits are a common feature across all aphasic clinical profiles and different patterns of performance and errors in lexical access across the two languages have been described in BWA (9, 10). The effectiveness of in-person speech and language therapy has been largely demonstrated in BWA (11–13), and semantic feature-based treatments targeting word retrieval deficits have often shown significant improvements in lexical access in one or both languages (14–17). However, access to appropriate clinical services for BWA may be often challenging for several reasons.

BWA may seek treatment in their native language because it is essential to communicate with others at home, especially if their degree of physical disability and limited independence after brain insult reduces their social interaction in the dominant language of their local environment (18). Also, bilingual clinical services that offer in-person treatment with the exact language combination demands of BWA may be limited or not readily available. Specialized bilingual outpatient rehabilitation services may be more frequently found in large urban settings (6, 19) compared with small cities or non-urban areas where specific bilingual groups may have lower demographic representation. Furthermore, the availability of trained professional interpreters and translators who assist non-bilingual clinicians might also be restricted to specific bilingual combinations and may vary according to service demand. Clinicians who are located within geographical reach but show low linguistic competency in the patient's two languages may find it difficult to work with BWA who show limited proficiency in the clinician's dominant language and provide appropriate assessments and treatment in the patient's other language (6, 20). Moreover, stroke is a leading cause of acquired disability (21), and stroke survivors present not only language impairments but also deficits in motor function, swallowing, vision, sensation, and cognition (22) that increase their difficulty in managing everyday activities, self-care independence, physical mobility, and participation in language therapy. In addition, several interacting factors that affect both rural and urban dwellers including geographical distance, travel time, transportation, related costs, and availability of caregivers to help patients attend in-person therapy sessions may further minimize the possibilities of BWA to access appropriate rehabilitation services and receive the language therapy they need. Importantly, continued access to clinical rehabilitation services is essential for people with chronic aphasia and reduced availability and limited access to bilingual clinical services contribute to poor access to health care in bilingual populations (23), which in turn reflects health care disparities that translate into reduced health care outcomes (6).

Telerehabilitation offers a promising approach for delivering language therapy to BWA as it enables the efficient use of rehabilitation resources while overcoming access barriers related to travel distance and shortage of bilingual clinicians for underserved populations (24). In particular, the use of videoconference and Internet-based customized therapy resources may facilitate the delivery of language rehabilitation by allowing clinicians to interact with their bilingual patients in real time, employing linguistically and culturally relevant therapy materials in the targeted language and measuring treatment outcomes in both languages. Research with monolinguals with aphasia addressing different language deficits and rehabilitation approaches has demonstrated the feasibility and benefits of telepractice interventions in this population (5). Moreover, some of these studies have demonstrated that teletherapy addressing word retrieval deficits can lead to significant improvements in lexical access in people with aphasia showing comparable results between videoconference and in-person delivery modalities (25–27). One of these studies has also provided evidence that treatment fidelity, a measure of the reliability with which therapy is consistently provided according to protocol, was equally high across treatment delivery modes (27). Notably, although the literature on teletherapy for bilingual aphasia is rather limited, a recent study with two Mandarin–English BWA has provided initial evidence of the effectiveness of therapy for lexical retrieval deficits delivered via videoconference (28). However, there is a paucity of research on the effectiveness of telerehabilitation for BWA and the reliability with which language interventions are implemented in this modality compared with in-person therapy for this population. Thus, more research is needed to determine whether treatment effectiveness and reliability is comparable across remote and face-to-face language therapy for BWA and to identify the benefits and challenges of teletherapy for this linguistically and culturally diverse population.

The aim of the present study was to determine whether the essential components of a semantic feature analysis treatment for word retrieval deficits in BWA could be delivered with equivalent effectiveness and reliability via telerehabilitation compared with in-person delivered therapy. To this aim, we contrasted (i) treatment effect sizes (ES) and change on secondary treatment outcome measures in the treated and the untreated language and (ii) treatment fidelity conducted by two trained independent bilingual raters on video-recorded treatment sessions across two patient groups, one receiving treatment via videoconference and the other receiving therapy in the in-person modality. We further evaluated inter-rater reliability (IRR) to identify the degree of agreement and consistency between raters on their judgment of clinicians' adherence to treatment procedures.

## Materials and Methods

### Study Design

Our telerehabilitation protocol is part of an ongoing prospective parallel-group, double-blind, phase II randomized controlled trial (RCT) (registered at www.ClinicalTrials.gov, identifier: NCT02916524) that aims to determine the capacity of our computational model BiLex (29) to predict language treatment outcomes in 48 Spanish–English BWA. Briefly, the RCT employs the BiLex model to simulate individual treatment outcomes in each language when treatment is provided in one language (e.g., English) vs. the other (e.g., Spanish). The comparison of these simulated effects allows the model to identify the optimal language for treatment (i.e., English or Spanish) that will lead to maximum therapy benefits across the two languages. Patients are randomly assigned to a model-prescribed experimental group receiving therapy in the optimal language as defined by the computational model, or to a model-opposite control group receiving therapy in the language not prescribed by the model. This randomized patient allocation will enable us to determine whether the computational model is able to identify the optimal language of treatment as reflected by the presence of superior treatment effects in the model-prescribed experimental group relative to the model-opposite control group. All patients receive the same semantic-based language treatment in English or Spanish, and patients who cannot attend in-person therapy sessions can receive therapy in the telerehabilitation modality. Language assessments (i.e., primary and secondary outcome measures) are conducted prior to and after treatment, and each patient receives both assessments and therapy in the same delivery modality (i.e., either in-person or via videoconference). As this is a double-blind RCT, both the researcher conducting the computational simulations that determine the optimal language of treatment for each patient and the clinicians conducting assessments and treatment are blind to each patient's group assignment. The study protocol of our RCT has been fully described and is available elsewhere (30).

It should be noted that the current study involves a retrospective analysis of patients who completed in-person therapy or telerehabilitation if they could not attend in-person assessment and treatment. Because the goal of the RCT is to determine whether the computational model is able to identify the optimal language for therapy, patients were randomized to a model-prescribed experimental group or to a model-opposite control group, instead of being randomly assigned to either treatment delivery modality (i.e., in-person vs. teletherapy). Both the in-person and remote treatments were administered as adherent to the protocol as possible, and the present study examined the effectiveness of teletherapy relative to in-person therapy, and the reliability with which essential treatment procedures were implemented across the two delivery modalities as evaluated by treatment fidelity scores.

### Participants

Participants were 16 Spanish–English bilingual speakers (six females, *mean age* = 56.93, *SD* = 17.31, *range* = 24.94–82.44 years; *mean number of educational years* = 14.56, *SD* = 3.08, *range* = 9–19) with chronic post-stroke aphasia (*mean time post stroke onset* = 69.27, *SD* = 104.45, *range* = 2.4–401.12 months). Participants were equally divided into a telerehabilitation group and an in-person therapy group to compare treatment effectiveness (i.e., eight patients in each group) and treatment reliability (i.e., six patients in each group)^1^. Groups were roughly matched by age, years of education, months post stroke onset, degree of aphasia severity, and overall naming ability in Spanish and English for both comparisons. The participants in the telerehabilitation group received therapy at home via videoconference because they could not attend in-person sessions due to geographic constraints and stroke-related difficulties. The participants in the in-person therapy group attended therapy sessions at one of the recruiting institutions pre-COVID19. In both cases, therapy was conducted by a bilingual clinician with training and experience in providing therapy using both delivery methods in accordance with our established RCT protocol (30). All participants had normal or corrected-to-normal vision and hearing, and demonstrated sufficient ability to understand and follow study procedures. None of them reported a history of psychiatric or neurological illness other than stroke. Participants were recruited via referrals from hospitals, bilingual research and rehabilitation centers, neurologists and speech and language pathologists, or via self-referrals across different locations in the United States including Massachusetts (*n* = 6), California (*n* = 4), Texas (*n* = 4), Rhode Island (*n* = 1), and Washington (*n* = 1). Table 1 summarizes the demographics of all the study participants.

**Table 1:** Demographic background of the bilingual adults with aphasia.

**Patient (sex)**	**Age**	**Education (years)**	**Months post onset**
**Telerehabilitation**			
P1 (F)	24.94	16	6.34
P2 (F)	47.2	19	53.15
P3 (M)	44.52	16	19.51
P4 (M)	70.49	12	6
P5 (M)	77.16	19	26.84
P6 (M)	62.73	10	23.85
P7 (M)	68.34	16	244.71
P8 (F)	78.47	11	38.53
**In-person therapy**			
P9 (F)	27.43	14	48.62
P10 (M)	39.65	13	40.34
P11 (F)	53.89	16	44.45
P12 (M)	82.44	16	401.12
P13 (M)	56.65	9	51.48
P14 (M)	69.31	12	10.32
P15 (F)	54.7	17	58.84
P16 (M)	53	17	37.88

### Ethics Statement

All procedures were reviewed and approved by the Boston University Charles River Campus Institutional Review Board at Boston, Massachusetts (reference number: 4492E). Participants provided their written informed consent to participate in our RCT and to be video-recorded during assessments and treatment regardless of treatment delivery modality.

### Assessment of Pre-stroke Bilingual Background

All patients completed the Language Use Questionnaire (31), which provides information about the age of acquisition of the patient's second language (L2) and different metrics known to influence prestroke proficiency in BWA (18) including language use, family proficiency, educational history, lifetime exposure, lifetime confidence, and self-ratings of language ability in English and Spanish (Table 2). *Age of acquisition* reflected the age of L2 learning onset. *Language use* measured the proportion of overall time participants and their conversation partners spent using each language on weekdays and weekends. *Family proficiency* evaluated the participants' ratings on their mother, father, and siblings' confidence in using each language expressed as an average proportion across family members. *Educational history* assessed the proportion of usage of each language by the participant and peers across different educational levels including elementary school, high school, and college. *Lifetime exposure* indicated the average proportion of time that participants heard, spoke, and read each language over their lifetime. *Lifetime confidence* measured the participants' average proportion of confidence in hearing, speaking, and reading each language over their lifetime. *Language ability rating* reflected the participants' average self-rated scores of prestroke ability to listen, speak, read, and write in Spanish and English. The LUQ administration was similar across delivery modalities. During in-person administration, the clinician gave the questionnaire to the patient and caregiver and asked them to provide information about the patient's bilingual background in each section described above, directly on the printed form. In the telerehabilitation modality, the clinician shared the LUQ form with the patient and caregiver over via videoconference, asked the questions included in each section of the questionnaire and wrote down their responses on the printed form.

**Table 2:** Prestroke bilingual background of the bilingual adults with aphasia as measured by the Language Use Questionnaire.

**Patient**	**L1^a^**							**L2**					
	**Use**	**Fam**	**Educ**	**Exp**	**Conf**	**LAR**	**AoA**	**Use**	**Fam**	**Educ**	**Exp**	**Conf**	**LAR**
**Telerehabilitation**													
P1	0.14	0.75	0.28	0.47	0.65	0.88	5	0.86	0.58	0.72	0.53	0.70	1
P2	0.09	1	1	0.60	1	1	18	0.91	0.17	0	0.40	0.45	0.91
P3	0.01	1	0.5	0.26	0.65	0.66	6	0.99	1	0.5	0.74	0.95	0.86
P4	0.70	1	0.75	0.81	1	1	15	0.30	0	0.25	0.19	0.48	0.88
P5	0.24	1	0.83	0.43	1	0.97	18	0.76	0.42	0.17	0.57	0.36	1
P6	0.66	0.92	0.83	0.67	1	1	16	0.34	0.12	0.17	0.33	0.42	0.83
P7	0.29	1	0.83	0.62	1	0.89	27	0.71	0.25	0.17	0.38	0.47	0.91
P8	0.45	1	0.58	0.54	1	1	10	0.55	0.25	0.42	0.46	0.51	0.97
**In-person therapy**													
P9	0.37	1	0.56	0.63	0.97	1	11	0.63	0.75	0.44	0.37	0.47	1
P10	0.35	1	0.94	0.76	1	1	21	0.65	0.17	0.06	0.24	0.44	1
P11	0.74	1	0.94	0.59	1	1	3	0.26	0.42	0.06	0.41	0.51	0.74
P12	0.62	1	0.89	0.80	1	1	35	0.38	0.17	0.11	0.20	0.36	0.8
P13	0.75	1	0.75	0.31	0.91	0.86	5	0.25	0.42	0.25	0.69	0.92	0.74
P14	0.25	0.92	0	0.50	0.81	1	3	0.75	0.83	1	0.50	0.97	1
P15	0.55	1	0.89	0.67	1	1	10	0.55	0.25	0.42	0.46	0.51	0.97
P16	0.02	1	1	0.68	1	1	12	0.98	0.08	0	0.32	0.46	1

a *Spanish was reported as the native language (L1) for most participants except for P9 (L1 = English). All metrics of bilingual history are expressed as proportions of time spent using a language in a given context (use, lifetime exposure, education history), ability (family proficiency, language ability rating), or confidence (lifetime confidence). Age of acquisition is expressed in years*.

*L1, native language; L2, second language; Fam, Family history; Educ, Educational history; Exp, lifetime exposure; Conf, lifetime confidence; LAR, language ability rating; AoA, Age of acquisition*.

### Language Assessments

Assessments were completed following our RCT protocol (30). All participants underwent a comprehensive battery of multiple tests, which were administered separately for each language on alternating English-only and Spanish-only testing sessions to avoid interference between languages. The present study reports the most relevant assessments for the clinical characterization of aphasia and language-processing abilities in our sample. Aphasia severity was determined using the English and Spanish versions of the Western Aphasia Battery—Revised (WAB-R) (32, 33) for patients assessed in person and the validated version of the WAB-R for videoconference (34) for patients in the telerehabilitation modality. Naming ability was assessed using the English and Spanish versions of the Boston Naming Test (BNT) (35, 36) and a 60-item naming screener developed in our laboratory (29), which required patients to name picture exemplars of high-frequency words presented on Microsoft PowerPoint in both languages. Non-verbal semantic knowledge was evaluated with the picture modality of the Pyramids and Palm Trees Test (PAPT) (37). Clinicians followed the standard administration procedures of these tests for patients in the in-person therapy group. For the telerehabilitation group, the clinician shared the test pictures via videoconference and asked the patient to either name the pictures shown on the computer screen (i.e., BNT, 60-item naming screener) or use the mouse to point to the bottom picture that was more related to the top picture (i.e., PAPT). Table 3 summarizes the clinical aphasia profile and individual scores of our patients on these language assessments prior to and after therapy.

**Table 3:** Profile of pre and post treatment scores on secondary treatment outcome measures in the treated and the untreated language in the bilingual adults with aphasia.

	**Treated language**						**Untreated language**						**NV**	
	**Aphasia profile (WAB AQ)**		**BNT**		**60-Item naming screener**		**Aphasia profile (WAB AQ)**		**BNT**		**60-Item naming screener**		**PAPT**	
**Patient**	**Pre**	**Post**	**Pre**	**Post**	**Pre**	**Post**	**Pre**	**Post**	**Pre**	**Post**	**Pre**	**Post**	**Pre**	**Post**
**Telerehabilitation**														
P1	Broca (37.3)	**Broca (56.8)**	17	13	17	DNT	Broca (27.3)	**Broca (28.7)**	1	1	0	DNT	42	DNT
P2	Anomic (79.1)	**Anomic (79.9)**	38	36	51	50	Broca (54.4)	**Broca (56.9)**	8	**11**	24	17	49	**51**
P3	Anomic (84.5)	Anomic (84.5)	28	28	34	33	Anomic (89.8)	**Anomic (93.5)**	47	45	47	47	47	**51**
P4	Conduction (57.3)	**Anomic (71)**	22	**26**	31	**37**	Broca (39.8)	**Broca (41.7)**	5	**10**	5	1	48	46
P5	Broca (67.4)	**Conduction (71.7)**	31	31	45	**47**	Broca (64.7)	**Conduction (78.6)**	30	**33**	37	**46**	50	49
P6	Broca (9.6)	Global (7.8)	0	0	0	0	Broca (10.8)	Global (5.2)	0	0	0	0	36	33
P7	Anomic (76)	**Anomic (83.8)**	31	30	40	**45**	Conduction (71.3)	**Conduction (78.8)**	24	17	26	**31**	48	**49**
P8	Conduction (76.8)	**Conduction (80.6)**	24	23	38	**40**	Anomic (78.9)	Anomic (78.8)	27	23	40	37	48	48
**In-person therapy**														
P9	Anomic (72.3)	**Anomic (74.1)**	14	13	27	23	Broca (66.4)	**Conduction (69.8)**	9	**15**	26	20	42	**43**
P10	Broca (39.5)	Broca (32.9)	3	**4**	11	**15**	Broca (21)	**Broca (32.8)**	4	4	2	**7**	46	45
P11	Conduction (68.8)	**Anomic (79.4)**	24	**26**	30	30	Anomic (90)	**Anomic (93.2)**	54	**55**	56	**59**	51	50
P12	Broca (55.7)	**Broca (56.8)**	8	6	21	DNT	Global (29.6)	**Global (31.9)**	1	1	2	DNT	22	DNT
P13	Anomic (91)	**Anomic (94.3)**	48	48	55	**56**	Anomic (83.2)	**Anomic (89.8)**	18	**26**	29	**31**	48	48
P14	Conduction (46.5)	Conduction (43.3)	11	**15**	6	**17**	Wernicke (33.9)	**Wernicke (36)**	6	6	3	**5**	48	48
P15	Anomic (74.1)	Anomic (74)	22	**28**	19	**35**	Broca's (68.5)	**Anomic (78)**	23	**26**	23	**42**	45	**47**
P16	Wernicke's (51.3)	**Wernicke's (53.9)**	13	**16**	15	**22**	Wernicke's (47.5)	**Wernicke's (53.8)**	4	**12**	11	**19**	46	**48**

*Improvement in scores from pre to post-treatment assessments are marked in bold*.

*NV, non-verbal testing; WAB-R AQ, Western Aphasia Battery-Revised Aphasia Quotient (max. score = 100); BNT, Boston Naming test (max. score = 60); PAPT, Pyramids and Palm Trees (max. score = 52); DNT, Did not test; Pre, Pre-treatment assessment; Post, Post-treatment assessment*.

### Stimuli

All patients completed the Item Selection Naming Test (ISNT), an extensive picture naming screener developed in our laboratory including 273 words across 13 broad semantic categories with validated semantic features (38). The test was created on Microsoft PowerPoint, and it was administered in each language separately in person or via videoconference following the same procedures described above for other picture naming tests. The test was used to identify items that each patient failed to name correctly in both languages. These items were used to create six 15-word sets including treatment words, untreated semantically related words, and control items in the language chosen for therapy, and their corresponding sets of translations in the untreated language. All six word sets were included in naming probes administered before, during, and after treatment to evaluate primary treatment outcomes in both languages. A detailed description of the naming probes and related procedures is available elsewhere (30).

### Treatment

All patients received therapy in one language targeting critical semantic features of the targeted trained items (16, 30), which entails retrieving the critical semantic features of the objects targeted in therapy. Treatment comprised 20 sessions in total (i.e., 2-h sessions twice per week). Patients received treatment in person if they were able to attend one of the main recruitment centers, or via videoconference if they were unable to complete in-person sessions due to geographical distance or any other stroke-related difficulties. The protocol was identical whether the treatment was administered in person or remotely, with the adjustments made to remote treatment described later in the Methods section.

All items from the ISNT that could be potentially selected as treatment words in either language, had a maximum of 24 semantic features that were validated for another study (36) by requesting healthy participants to decide whether or not a given feature (e.g., “can fly”; “is a household item”) applied to a given word picture exemplar (e.g., “vulture”). The percentage of healthy adults who considered a semantic feature as being applicable or not applicable to a given word during feature validation determined how the feature would be addressed in treatment (i.e., features were treated as applicable if more than 50% of healthy individuals rated it as applicable). In this way, the validation of semantic features helped clinicians to guide patients on treatment steps that involved the classification and verification of semantic features for treated words (see Treatment Steps later in this section).

### Technical Requirements, Software, and Setup

**Figure 1:**
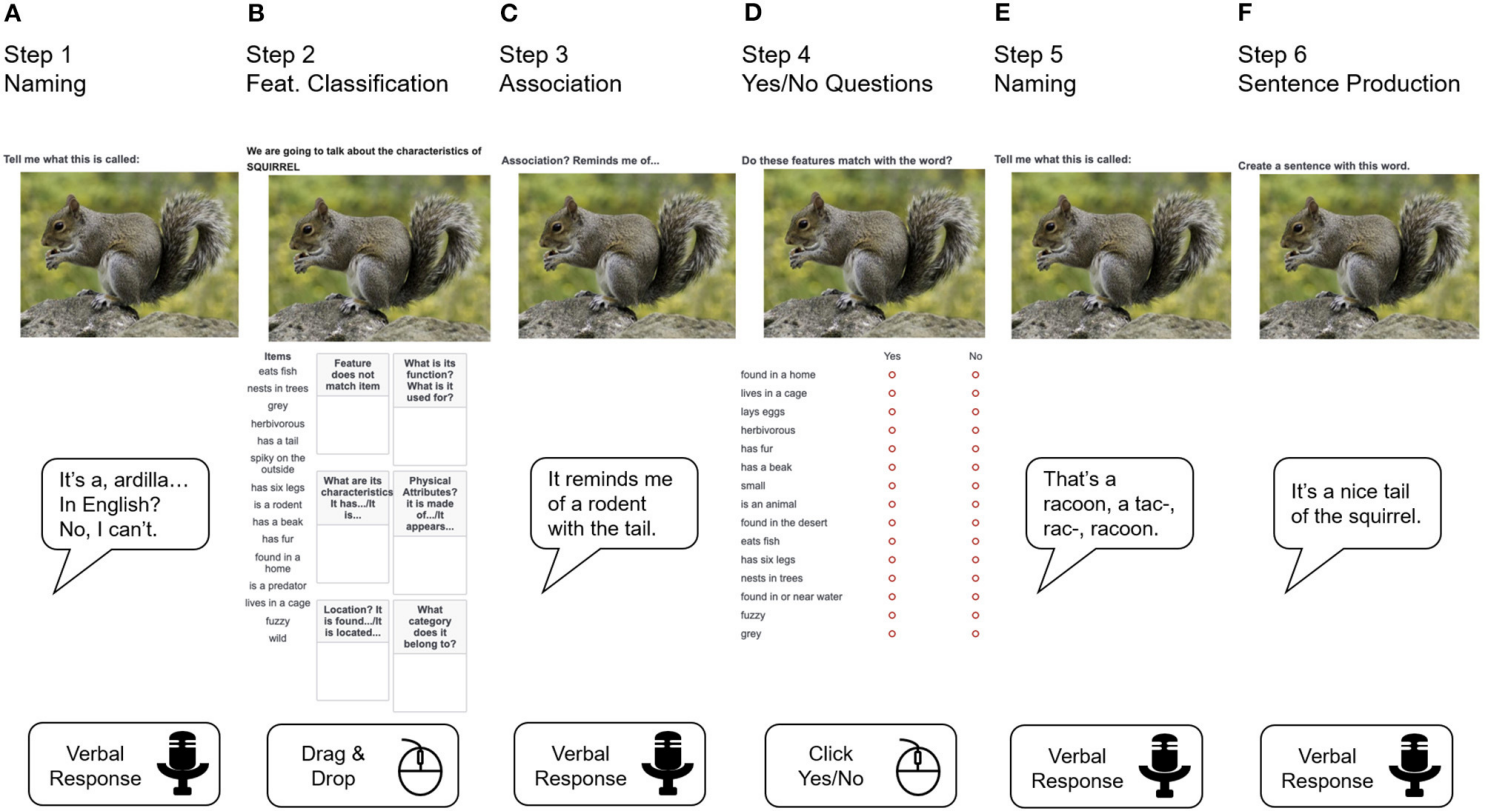
Treatment steps. Example of treatment steps presented across six consecutive screen displays for the treatment item “squirrel.” In step 1, the patient provides a verbal naming response for the picture **(A)**. In step 2, the patient drags and drops the semantic features that do not apply to the pictured item into the “feature does not match item” box with the mouse (step 2A) and assigns the remaining features to the other boxes (step 2B) **(B)**. In step 3, the patient provides an association for the item **(C)**. In step 4, the patient decides whether each semantic feature matches the item by clicking on the yes/no options with the mouse **(D)**. In step 5, the patient completes a second naming trial for the picture **(E)**. In step 6, the patient generates a sentence using the treatment word **(F)**.

In both delivery modalities, treatment was conducted on a laptop or desktop computer using the Internet-based Qualtrics survey software available at https://www.qualtrics.com. Twenty Qualtrics surveys were developed for each patient, one per treatment session. Each survey presented 15 treatment items in randomized order in the language chosen for treatment, with one treatment step displayed on the screen at a time (Figure 1). Surveys were presented online on the Google Chrome web browser using the Zoom communication software available at https://zoom.us/ to enable video recording of all treatment sessions for reliability analyses and offline scoring of patient responses across treatment steps^2^. The standard Internet connection required followed the Zoom videoconference standard specifications (i.e., broadband wired or wireless 3G or 4G/LTE, with a minimum bandwidth of 600 kbps for up/down). The computer setup required a mouse, microphone, speakers, and a webcam, which could be either a USB plug-in, wireless Bluetooth, or built into the computer. The clinician used an additional computer to access the patient's treatment key designed to provide accuracy feedback for patients' responses in each treatment step, to annotate all verbal responses during therapy, and to score patient responses offline once therapy was completed.

### Treatment Steps

In both delivery modalities, a clinician guided each patient throughout six treatment steps emphasizing the semantic feature attributes of each treated item (Figure 1). Patients were encouraged to provide responses for each treatment step and were allowed to make corrections to their own responses. No feedback on response accuracy was provided until a final response was obtained from the patient, and only then were responses considered for scoring prior to clinicians' feedback. The six treatment steps were provided as follows.

In step 1 *Naming*, a picture of the treatment item was shown on the screen. Next, the clinician asked the patient to name the item, typed down the response verbatim in the patient's treatment key, and provided verbal feedback (i.e., correct response) regardless of response accuracy. In step 2 *Feature classification*, the treatment item was shown together with a list of 15 semantic features randomly retrieved from a pool of a maximum of 24 features that either applied or did not apply to that item. In step 2A *Feature selection*, the patient was asked to review each feature in the list and transfer the ones that did not apply to that item into the box “feature does not match item” using the computer mouse. Once this step was completed, the clinician provided feedback by explaining why misclassified features required correction and rearranging them such that only the features that applied to the treated item remained in the feature list. In step 2B *Feature assignment*, the patient was asked to classify the features that applied to the treatment item into one of five boxes (i.e., function, characteristics, physical attributes, location, and superordinate category) where they best fit. Once this section was completed, the clinician provided feedback by explaining why misclassified features would best fit a different box and rearranging them into the correct boxes. The survey allowed for clicking on the written features to hear them aloud before moving them into the boxes, which was particularly helpful for patients with reading difficulties. In step 3 *Association*, the treatment item was shown again and the patient was requested to think of something else it reminded them of, or something it was associated with. If the association was not immediately clear, the clinician requested the patient to explain the association and recorded the response and explanation verbatim into the treatment key. In step 4 *Yes/No questions*, the treated item was shown with a list of 15 semantic features randomly retrieved from the entire pool of features validated for that item. The patient was asked to decide whether or not each feature matched that particular item by clicking the “yes” or “no” response options with the computer mouse. This allowed the patient hearing the chosen response for that particular feature read aloud (e.g., *YES to “made of fabric*”). Once all the responses were registered, the clinician provided feedback by making corrections to all incorrect responses and providing an explanation for them. In step 5 *Naming*, the patient saw the picture of the treated word again and was requested to name it. The clinician interventions were similar to step 1. In step 6 *Sentence production*, the patient was asked to create a short sentence with the trained word. Next, the clinician typed down the response verbatim in the treatment key and provided feedback by correcting the sentence into a grammatically and semantically acceptable sentence with the target word.

### Treatment Key

**Figure 2:**
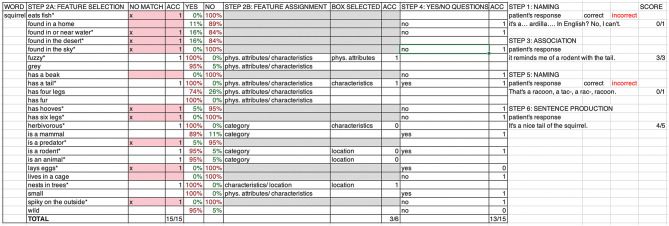
Treatment key. Example of a treatment key displaying the treatment word “squirrel” (column 1). The key helps clinicians to document and score patient responses and provide feedback across treatment steps. The 15 out of 24 semantic features randomly presented in a session are marked with an asterisk (column 2). Features that do not apply to the item are color marked according to the percentage of individuals who determined the feature to be applicable or non-applicable during feature validation (columns 5–6). Patient motor responses are collected for treatment steps 2 (columns 3 and 8) and 4 (column 10), and scored for accuracy (ACC; columns 4, 9, and 11) following color-coded indications on whether or not the feature applies to the item (column 3 for step 2A and columns 7–9 for step 4) or the correct response key for feature assignment (column 7). Verbal responses generated during treatment steps 1, 3, 5, and 6 are written down verbatim by the clinician during the session (column 12).

An individual treatment key was generated for each patient on a Microsoft Excel spreadsheet consisting of 20 treatment tabs (one per treatment session) including the sequential presentation of all 15 treatment items within each treatment session. Figure 2 depicts an example of a treatment key with one treatment item for a patient receiving therapy in English. For each treatment item, the treatment key provided the clinician with (i) a list of all the validated semantic features (up to 24 attributes available per item), (ii) a color-coded indication as to whether each feature should be considered applicable or non-applicable for that particular item on the basis of the feature validation ratings mentioned above (treatment steps 2 and 4), (iii) a correct response key to classify each applicable feature as belonging to one or more feature classification boxes including function, characteristics, physical attributes, location, and category (treatment step 2), and (iv) specific fields to manually input the feature classification box selected by the patient (treatment step 2), answers to Yes/No questions (treatment step 4), and patient responses to treatment steps involving open questions (treatment steps 1, 3, 5, and 6). In each session, clinicians typed the patient's verbal responses verbatim into the treatment key while performing each treatment step. The scoring of the patient's performance was conducted offline by trained research assistants reviewing videotaped recordings of each session.

### In-person Therapy Administration

Both clinician and patient sat in front of a laptop computer in a clinical consulting room at one of the recruitment institutions. First, the clinician started a new session on Zoom and verified that the volume, speakers, front camera, and mouse were working properly. The patient was reminded that the session would be recorded, and the clinician activated the “record” function on Zoom to start video recording the session. Next, the clinician activated the “share screen” function on Zoom to display the Qualtrics survey containing the corresponding treatment session and conducted all treatment steps while recording the patient's verbal and motor responses. The clinician started guiding the patient through all treatment steps (Figure 1) while also accessing the treatment key of the corresponding session (Figure 2) on another laptop computer to provide accuracy feedback and to input the patient's verbal responses. If the patient was not comfortable using the mouse or was unable to handle it due to hemiparesis, the clinician could control the mouse to generate the patient's response choice for treatment steps that required a motor response. For instance, the clinician could ask the patient to verbally indicate whether or not a given feature should be moved into the “does not apply to this item” box or point to one of the five feature classification boxes where a given applicable feature would best fit it so that the clinician could drag and drop the feature into the selected box (treatment step 2). The clinician could also ask the patient whether or not a feature applied to a treatment item and use the mouse to select the “Yes” or “No” response options for the patient (treatment step 4). Once the session was completed, the recording was stopped and downloaded onto a local computer.

### Videoconference Therapy Administration

Clinicians first determined whether a patient would be eligible to receive telerehabilitation at home by requesting information from the patient and caregivers about access to a computer and Internet connection, and patient's ability and comfort with using the computer independently for therapy at home. If the patient was not sufficiently independent to use the computer, clinicians further asked whether the patient's caregiver could help them set up the computer and connect to the Zoom meeting for every treatment session. Patients who did not have a computer at home were offered the possibility of borrowing all the necessary equipment from our laboratory. In this case, the caregiver would be requested to collect the equipment in person, fill out and sign a checkout form confirming that they would return the equipment at the end of the patient's participation in our study.

Patients who felt confident using the computer and had enough control of the mouse to provide motor responses were sent an email with an invitation to join the Zoom videoconference session and a link to the Qualtrics survey with the appropriate treatment session so that they could set up the Zoom connection and the Qualtrics treatment survey independently. Once connected to the session, the clinician indicated that the video recording would start immediately and requested the patient to share access to their computer screen. This enabled the clinician to see both the participant via the front camera of the computer, and the Qualtrics survey on the participant's computer. The clinician guided the patient through treatment steps while accessing the treatment key on another laptop computer to type down the patient responses and provide feedback. If at any point the patient required assistance, the clinician could request the patient to activate the “remote control” function of Zoom to gain access to the patient's screen and handle the mouse remotely, either to troubleshoot any difficulties or to generate responses for treatment steps 2 and 4 after asking the patient for his or her response choice. The Qualtrics survey had a pre-determined expiration time so that patients with a direct link to the survey would not attempt additional practice once the treatment session was completed.

For patients for whom independent use of the computer was not possible due to motor difficulties or lack of confidence with computer and Internet use, the clinician sent a link to the patient or caregiver via email to join the Zoom videoconference session and opened the Qualtrics survey for the corresponding treatment session directly on his or her local computer. The clinician could then share the computer screen to make the survey visible to the patient, start recording the session, guide the patient across treatment steps, and generate the motor responses for treatment steps 2 and 4 according to the patient response choices while also having the patient visible via the front-camera. Thus, treatment was kept similar across both delivery methods with the only difference that patients in the telerehabilitation modality would receive therapy at home while being connected over videoconference.

### Data Management and Confidentiality

Qualtrics surveys collected de-identified data from patients. Zoom video recordings were directly downloaded on the local computer after each session, and they were immediately transferred to the laboratory server, which only the researchers in the study could access. All other personal information and assessment and treatment data were stored and managed using REDCap, a secure web-based software platform designed to support data capture for research studies (39). Qualtrics, Zoom, and REDCap were hosted at Boston University. To ensure privacy and confidentiality, patient electronic data were kept in password-protected computer files, while paper forms and other study materials were kept in physical folders stored in a locket cabinet at Boston University.

### Treatment Effectiveness

Treatment effectiveness was evaluated by computing ES for direct treatment effects (i.e., trained items in the treated language) and indirect treatment effects (i.e., untrained translations in the untreated language). ES is a standard measure of the extent to which changes from baseline to after treatment in primary treatment outcomes (i.e., naming probes) are statistically reliable. ES were computed as [(mean of post-treatment probes – mean of baseline probes)/standard deviation of baseline], and defined as small (4.0), medium (7.0), and large (10.1) ES according to the benchmarks proposed for treatments focused on lexical retrieval (40). In addition, to evaluate treatment effects on secondary outcome measures (i.e., standardized language assessments), we computed treatment-related change scores (post-treatment score – pre-treatment score) for each patient on the WAB-AQ, BNT, 60-item naming screener, and the PAPT in the treated and the untreated language separately.

### Treatment Reliability

Treatment fidelity was the measure employed to assess the reliability of the administration of treatment in each modality and the equivalence of procedures delivered across in-person therapy and telerehabiliation. This comparison is important because clinician's behavior during therapy may differ between the remote and face-to-face settings. Treatment fidelity was conducted by two independent fluent English–Spanish bilingual research assistants who used a treatment fidelity scoring form developed for this study to assess clinicians' adherence to treatment procedures (Supplementary Materials). The fidelity assessment focused on evaluating the clinicians' behavior during therapy using a specific scoring system that determined whether specific procedures involved in each treatment step were delivered as planned (1 point = fully delivered, 0.5 points = partly delivered, 0 points = not delivered). The scoring system allowed raters to provide partial credit for procedures that were not fully delivered in steps 2 and 4, which required multiple clinician–patient interactions, whereas all other steps could be credited 1 or 0 points as clinician–patient interactions were shorter, and procedures were more straightforward.

The two independent raters received 8 h of training to conduct treatment fidelity assessments. Training included a detailed revision of (i) the manual of treatment steps for clinicians according to our RCT protocol (30), (ii) the treatment fidelity scoring form to evaluate clinicians' adherence to protocol across all six treatment steps, (iii) a troubleshooting form including scoring examples of interventions made by clinicians across treatment steps, and (iv) supervised treatment fidelity scoring of two treatment videos (i.e., four treatment items in total) of two participants included in the RCT but not reported here. In addition, as part of the final calibration step of training, each rater independently scored two 2-h videotaped treatment sessions of these two participants, resolved potential discrepancies between each other in their scoring, and received feedback on their final ratings.

Once training was completed, treatment fidelity was conducted for both treatment delivery modalities separately. Each rater independently reviewed and rated clinician's adherence to treatment steps for six patients (telerehabilitation *n* = 3, in person therapy *n* = 3) on 25% of their videotaped treatment sessions (i.e., five randomly selected videos out of 20 treatment sessions per patient, 30 2-h treatment sessions per rater in total). Treatment fidelity was computed as the percentage of points obtained by clinicians for adherence to protocol procedures across all treatment steps evaluated across the five treatment sessions per patient. Treatment fidelity for the in-person vs. the telerehabilitation modality was then compared using independent samples *t*-tests. Finally, assessing inter-rater reliability (IRR) allows quantifying the degree of agreement and consistency between trained coders who provide independent observation ratings for a set of collected data (41) and is a suggested benchmark to evaluate aphasia treatment fidelity procedures (42). In order to evaluate the degree to which scores were consistent between raters, each independent rater additionally scored 20% of all video recorded treatment sessions initially reviewed by the other rater. IRR was then assessed using two-way mixed, average measures, intraclass correlations (ICCs) for absolute agreement, and we used established cutoffs as reference for the qualitative interpretation of IRR (43) considering it to be poor for values <0.40; fair for values between 0.40 and 0.59, good for values between 0.60 and 0.74, and excellent for values between 0.75 and 1.0.

## Results

### Between-Group Comparisons on Demographic and Clinical Variables

Patients in the telerehabilitation group (*n* = 8) did not significantly differ from the in-person therapy group (*n* = 8) in terms of their age [telerehabilitation: *M* = 59.23, *SD* = 18.71; in-person therapy: *M* = 54.63, *SD* = 16.73; *t* (14) = 0.518, *p* = 0.612], number of years of education [telerehabilitation: *M* = 14.88, *SD* = 3.48; in-person therapy: *M* = 14.25, *SD* = 2.82; *t* (14) =.395, *p* =.699], months poststroke onset [telerehabilitation: *M* = 51.92, *SD* = 79.59; in-person therapy: *M* = 86.63, *SD* = 127.88; *t* (14) = −0.652, *p* = 0.525], aphasia severity as measured by the WAB-AQ scores in English [telerehabilitation: *M* = 55.69, *SD* = 25.32; in-person therapy: *M* = 59.87, *SD* = 22.86; *t* (14) = −0.347, *p* = 0.734] and Spanish [telerehabilitation: *M* = 60, *SD* = 27.41; in-person therapy: *M* = 57.22, *SD* = 20.55; *t* (14) = 0.229, *p* = 0.822], naming ability as measured by the BNT scores in English [telerehabilitation: *M* = 19.37, *SD* = 15.29; in-person therapy: *M* = 19.12, *SD* = 20.88; *t* (14) = 0.027, *p* = 0.979] and Spanish [telerehabilitation: *M* = 22.25, *SD* = 14.16; in-person therapy: *M* = 13.62, *SD* = 7.37; *t* (14) = 1.528, *p* = 0.149], or naming ability as measured by the 60-item naming screener in English [telerehabilitation: *M* = 24.25, *SD* = 16.40; in-person therapy: *M* = 23.75, *SD* = 21.16; *t* (14) = 0.053, *p* = 0.959] and Spanish [*M* = 30.12, *SD* = 19.58; in-person therapy: *M* = 18.25, *SD* = 10.99; *t* (14) = 1.496, *p* = 0.157] (the same between-group comparisons yielded *p* values ≥ 0.237 in all cases when only considering the 12 patients included in the statistical analyses comparing treatment reliability between the two delivery modes). These comparisons confirm that the in-person and the telerehabilitation groups were comparable on critical demographic and clinical variables that may influence between-group differences in our treatment effectiveness and reliability analyses.

### Treatment Effectiveness Across Delivery Modalities

**Figure 3:**
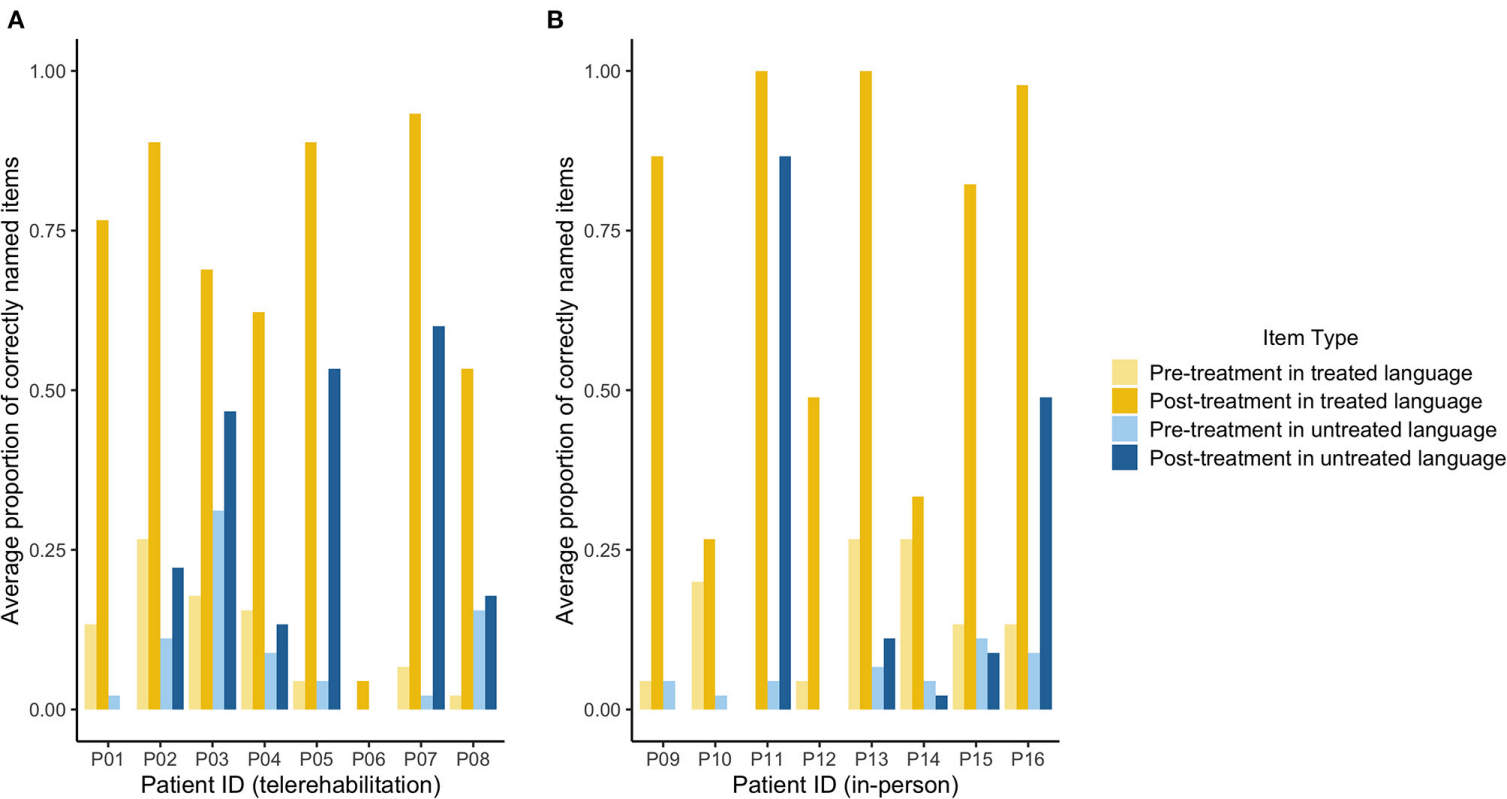
Pre and post treatment naming scores for the telerehabilitation group and the in-person therapy group. The average proportion of correctly named items across three naming probes is shown for treated items (yellow) and untreated control items (blue) prior to treatment (lighter colors) and after treatment (darker colors) for each patient in the telerehabilitation group **(A)** and the in-person therapy group **(B)**.

The results of treatment effectiveness are shown in Table 4 and Figure 3. ES computed for 15 patients was used for the between-group comparisons of treatment effects reported in this section (ES could not be computed for P6 due to extremely low accuracy in naming probes). The evaluation of direct treatment effects indicated that 13 out of 15 patients demonstrated significant improvement on trained items in the treated language (i.e., ES > 4.0), with three patients showing medium ES (i.e., ES > 7.0) and 10 patients showing large ES (i.e., ES > 10.1). We found no significant differences in ES for treated items in the treated language between the telerehabilitation group (*M* = 14.57, *SD* = 5.48) and the in-person therapy group (*M* = 11.78, *SD* = 8.62) [*t* (13) = 0.734, *p* = 0.476]. The assessment of indirect treatment effects revealed that five out of 15 patients showed significant improvement on translations in the untrained language (i.e., ES > 4.0), with one patient showing a small ES (i.e., ES > 4.0) and four patients showing a large ES (i.e., ES > 10.1), thus showing evidence of cross-language generalization effects in the present sample. Again, there were no significant differences in ES for translations in the untreated language between the telerehabilitation group (*M* = 5.11, *SD* = 6.19) and the in-person therapy group (*M* = 3.79, *SD* = 8.04) [*t* (13) = 0.353, *p* = 0.73]. ES for untrained control items was minimal for most patients in the telerehabilitation and the in-person groups and were within the range of ES reported in previous treatment research with BWA using the same semantic-based intervention (16). Only one patient in the telerehabilitation group showed a medium ES for untreated control items in the treated language and a small ES for their corresponding translations, and two patients in the in-person therapy group showed either a small ES for untreated control items in the treated language or a medium ES for untreated control items in the untreated language (Table 4). There were no significant differences in ES for control items in the treated language between the telerehabilitation group (*M* = 2.81, *SD* = 2.95) and the in-person therapy group (*M* = 1.43, *SD* = 2.26) [*t* (13) = 1.029, *p* = 0.322] or in ES for their corresponding translations between the telerehabilitation group (*M* = 0.62, *SD* = 2.07) and the in-person therapy group (*M* = 1.49, *SD* = 2.61) [*t* (13) = −0.711, *p* = 0.49].

**Table 4:** Treatment effectiveness as measured by effect sizes (ES) for the treated language and the untreated language across delivery modalities.

**Patient**	**Treated language**	**ES treated language (treated items)^a^**	**ES treated language (control items)^a^**	**Untreated language**	**ES untreated language (translations-treated items)^a^**	**ES untreated language (translations-control items)^a^**
**Telerehabilitation**						
P1	English	9.5	2.51	Spanish	−0.58	0
P2	Spanish	9.33	2.31	English	2.89	0
P3	Spanish	13.28	−1.15	English	4.04	−2.31
P4	Spanish	12.12	1.6	English	1.15	1.73
P5	Spanish	21.94	8.66	English	12.70	4.04
P6	English	NA^b^	NA^b^	Spanish	NA^b^	NA^b^
P7	Spanish	22.52	2.31	English	15.01	1.73
P8	English	13.28	3.46	Spanish	0.58	−0.87
**In-person therapy**						
P9	Spanish	21.36	−2.31	English	−1.15	−0.58
P10	English	1.73	−1.15	Spanish	−0.58	1.15
P11	Spanish	25.98	3.46	English	21.36	1.73
P12	Spanish	11.55	1.15	English	0	0
P13	English	11.00	4.62	Spanish	1.15	1.73
P14	English	1.00	2.31	Spanish	−0.58	−1.15
P15	Spanish	8.95	1.73	English	−0.29	1.73
P16	Spanish	12.67	1.6	English	10.39	7.33

a *Effect sizes defined as small (ES > 4.0), medium (ES > 7.0), or large (ES > 10.1 = large) according to the benchmarks proposed for treatments focused on lexical retrieval (40)*.

b *NA, Not available. Calculation of ES was not possible for P6 and therefore, this participant was excluded from statistical analyses*.

All patients except for P6 also showed improvement in at least one secondary treatment outcome measure (Table 3). Independent-sample *t*-tests were conducted to assess differences between the telerehabilitation group and the in-person therapy group on treatment-related change scores (post-treatment score – pre-treatment score) on secondary treatment outcome measures (i.e., WAB-AQ, BNT, 60-item naming screener, and the PAPT). As shown on Table 5, there were no significant differences on treatment-related change scores on secondary treatment outcome measures between groups according to treatment delivery modality (all *p*-values ≥ 0.106).

**Table 5:** Comparisons between the telerehabilitation and the in-person therapy groups on treatment-related change scores on secondary outcome measures in the treated and the untreated language.

**Secondary outcome measure**	**Telerehabilitation**	**In-person therapy**	**Between-group comparison**
WAB-AQ (treated language)	5.54 ± 8	1.19 ± 5.03	*t* (14) = 1.302, *p* = 0.214
WAB-AQ (untreated language)	3.62 ± 4.97	5.65 ± 3.56	*t* (14) = −0.936, *p* = 0.365
BNT (treated language)	−0.5 ± 2.27	1.62 ± 2.67	*t* (14) = −1.716, *p* = 0.108
BNT (untreated language)	−0.28 ± 4.31	3.25 ± 3.57	*t* (13) = −1.738, *p* = 0.106
60-item naming screener (treated language)	1.86 ± 2.79	5 ± 6.88	*t* (12) = −1.12, *p* = 0.285
60-item naming screener (untreated language)	0 ± 5.48	4.71 ± 7.61	*t* (12) = −1.330, *p* = 0.208
PAPT	0.14 ± 2.41	−0.43 ± 1.27	*t* (12) = −0.277, *p* = 0.786

*Treatment-related change scores were computed as post-treatment score–pre-treatment score on each secondary treatment outcome measure in the treated and the untreated language. WAB-R AQ, Western Aphasia Battery-Revised Aphasia Quotient; BNT, Boston Naming test; PAPT, Pyramids and Palm Trees*.

### Treatment Reliability Across Delivery Modalities

The results of the treatment fidelity ratings are shown in Table 6. The difference between the average percentage of treatment steps correctly conducted by clinicians according to protocol per patient in the telerehabilitation modality (*M* = 98.73%, *SD* =.61%) and in the in-person modality (*M* = 97.54%, *SD* = 2.56%) was statistically non-significant [*t* (10) = 1.103, *p* = 0.296]. IRR analyses assessing the extent to which our two independent raters agreed on their judgment of clinician's adherence to treatment procedures in each delivery modality further revealed similarly high ICC values of 0.990 [95% CI (0.937–0.999)] for the telerehabilitation modality and of 0.997 [95% CI (0.983–1)] for the in-person modality indicating excellent agreement and high consistency between the two independent raters.

**Table 6:** Treatment fidelity across the telerehabilitation and the in-person therapy modalities.

**Patient**	**Rater**	**Number of session scored (1–20)**	**Treatment steps (max. score)^a^**	**Treatment steps (actual score)**	**%Steps correctly delivered**
**Telerehabilitation**					
P1	2	1–8–12–16–18	399	393.5	98.62
P2	2	1–6–10–14–16	476	471.5	99.05
P3	2	3–5–8–17–20	525	521	99.23
P4	1	1–4–9–14–19	186	183	98.38
P5	1	2–6–9–15–20	397	388	97.73
P6	1	4–9–12–15–20	154	153	99.35
**In-person therapy**					
P7	1	4–7–10–13–19	383	377	98.43
P8	2	8–10–12–16–17	195	192	98.46
P9	2	3–6–11–15–18	308	284.5	92.37
P10	1	3–7–11–13–19	329	327	99.39
P11	2	2–5–7–13–20	483	475	98.34
P12	1	3–5–11–14–17	342	336	98.24

a *The total number of scored treatment steps varied across patients despite keeping the number of treatment sessions constant (five sessions per patient) because the treatment was self-paced and each session covered as many treatment items as the patient was able to go through in each 2-h session*.

## Discussion

The present study aimed to evaluate treatment effectiveness and reliability in a videoconference-delivered semantic feature analysis intervention for word retrieval deficits in Spanish–English BWA compared with in-person delivered therapy, to establish the equivalence of treatment gains, and quality of the delivery of essential components of therapy across delivery modalities. In what follows, we discuss important aspects of the effectiveness and the reliability of telerehabilitation as a treatment delivery model for BWA, and the potential benefits and challenges evidenced in the conduct of this study.

Our study demonstrates that the treatment effects of teletherapy on both the treated and the untreated language are comparable to those observed in the in-person delivery modality while accounting for multiple factors that may influence individual variation in treatment outcomes in BWA. More specifically, direct treatment effects were evidenced by significant improvement on treated items, which achieved predominantly medium and large ES in the treated language for most patients across the two modes of therapy delivery. Cross-language generalization was also evidenced in both groups, although fewer patients demonstrated significant ES in the untreated language, and treatment effects also generalized to untreated control items in the treated or the untreated language for three patients across both delivery modalities. The only patient who did not show improvements in either therapy delivery method was P6, possibly because he was non-fluent in both languages and also showed the highest degree of aphasia severity after stroke. Overall, our findings align with previous research showing positive results for a variety of language interventions via teletherapy for adults with aphasia demonstrating equivalent treatment gains relative to in-person treatment (5) and provide further evidence for the effectiveness of semantic feature analysis-based treatments for word retrieval deficits in BWA (14, 16) regardless of delivery method.

The evidence of direct treatment effects on treated items described above was further supported by the presence of improvements in at least one other secondary treatment outcome measure including aphasia severity (i.e., WAB-AQ scores), naming ability (i.e., BNT and 60-item naming screener scores), and lexical–semantic knowledge (i.e., PAPT scores), which were observed in the treated and the untreated language in both groups. Moreover, treatment-related change scores (i.e., change on post-treatment relative to pre-treatment scores) on these secondary outcome measures did not differ significantly between groups in the treated or the untreated language. These results suggest that treatment effects on secondary outcome measures are comparable across treatment delivery modalities and suggest that far transfer to standardized tests may be possible in both languages subsequent to treatment of specific lexical items, although the extent of these effects may vary across individuals being more likely to occur in treatment responders (44). Thus, the fact that these language assessments were able to capture treatment-related change in both groups, suggests that they could be employed as reliable secondary outcome measures in telerehabilitation for bilingual aphasia.

An important aspect to consider when providing language therapy for BWA via telerehabilitation is the extent to which the main components of an intervention can be implemented with equal quality and accuracy relative to the standard in-person delivery approach. Our analysis of treatment fidelity conducted for both delivery methods showed high clinician adherence to treatment protocol for both delivery modalities and no significant differences in the percentage of correctly implemented treatment steps in the treatment sessions conducted with patients receiving telerehabilitation compared with those receiving in-person therapy. Furthermore, IRR was excellent for both telerehabilitation and in-person therapy, demonstrating high agreement between raters and consistency in their judgment of correct implementation of treatment procedures in the two service delivery modalities. These findings provide evidence that our semantic feature analysis-based treatment for word retrieval deficits in BWA can be reliably implemented by different clinicians via videoconference in a similar manner with comparable quality relative to in-person treatment. Our results are in line with prior research providing evidence that the reliability of treatment for word retrieval deficits in monolinguals with aphasia is similar across the remote and in-person delivery modes (27). Moreover, we suggest that both the development of detailed telerehabilitation treatment protocols and intensive training procedures for clinicians and treatment fidelity raters are crucial to ensure the effective implementation of the intended treatment components and a reliable assessment of treatment fidelity in clinical rehabilitation research. The evaluation of treatment fidelity is important to improve confidence in the findings of research involving behavioral interventions (45, 46), especially when treatment is provided using less common approaches or delivery methods. While it is important to assess whether clinicians who provide the same treatment to different individuals with aphasia do so in the same way to ensure the validity of the therapeutic effects (47), treatment fidelity has been evaluated inconsistently and infrequently in aphasia intervention studies and RCTs (48, 49), and only a limited number of studies have conducted treatment fidelity on telerehabilitation for adults with aphasia (27, 28). Thus, the present study contributes to the small but important number of studies documenting treatment fidelity in clinical aphasia research and RCTs, and underscores the importance of establishing whether clinician behavior is compliant with treatment protocol across different therapy delivery approaches.

Our study also provides evidence for the practicality and technical usability of teletherapy to deliver semantic feature analysis treatment for word finding deficits in BWA. The Zoom videoconferencing platform enabled clinicians to communicate with patients in real time, provide them with assistance during treatment, and have later access to good quality video-recorded treatment sessions. The Qualtrics software supported successful treatment delivery in the videoconference relative to the in-person modality and clinicians were satisfied with its usability in remote therapy. While Qualtrics was used in the context of synchronous teletherapy based on live clinician–patient interactions, it could also be employed in an asynchronous format based on the offline transmission of patient outcomes (5). The Qualtrics survey parameters are highly customizable in terms of the number of treatment items and semantic features that can be presented per session, which makes them suitable to send the patient home practice assignments and collect additional data on the patient's performance offline. Although not used in this study, its use in the asynchronous teletherapy format could allow examining if self-paced additional exposure and practice with treatment items can further enhance treatment benefits and assess the cost-effectiveness of self-managed computerized therapy (50). Also, the survey can adapt well to different devices including desktop, laptop computers, and tablets allowing patients the flexibility to use the device of their choice as done in other studies (27).

It is also important to consider a few potential implementation challenges for the delivery of semantic feature analysis treatment via telerehabilitation. For instance, patients may differ in the type and amount of assistance needed to access therapy online depending on the degree of their motor impairment. Furthermore, receptive language difficulties may impact the ability of people with aphasia to follow instructions for accessing teletherapy independently (51) making additional training necessary to employ the technology effectively (52). Our treatment setup minimized motor demands by allowing clinicians to facilitate patients' motor responses when needed. Most patients with mild to moderate impairments were sufficiently independent to follow instructions to start the videoconference connection and go through the treatment steps with only minimal remote control support by the clinician (e.g.,: P1, P2, P3, and P5). However, P4 and P6 were more severely affected and needed the support of the clinician and the caregiver, respectively, to set up the computer and videoconference session and complete treatment procedures. Thus, it is possible that videoconference therapy is not fully suitable for patients with severe language and/or motor difficulties, limited experience with technology, and lack of caregiver support. Age is another factor that can negatively impact Internet use, computer literacy, and acceptability of new technology (53, 54). Although we did not conduct a patient satisfaction and acceptability survey after participation, the interaction of older patients with clinicians went smoothly, and all patients regardless of age showed high adherence to treatment, having completed all sessions as planned. It is possible that computer and Internet use via a proxy helped our older and less independent patients gain confidence in this method of treatment delivery and focus on language treatment goals instead of achieving independent computer use. Overall, our findings support the implementation of language therapy for individuals with aphasia in the telerehabilitation modality as shown in previous studies (27, 51, 55, 56), and suggest that videoconference and customized, Internet-based software can facilitate the delivery of semantic feature analysis treatment for BWA via telerehabilitation.

The present study has important implications for bilingual aphasia research and practice. As telerehabilitation is an emerging research field, effectiveness and reliability studies are essential to demonstrate that specific language interventions can be successfully delivered via telerehabilitation and support its potential to overcome access difficulties to bilingual clinical services for BWA. An important goal in bilingual aphasia rehabilitation is to provide optimal therapy for existing language deficits while considering the patient's bilingual background, impairment in the two languages, and patient and family communication needs. However, considering all of these factors in treatment planning for BWA might be restrictive in contexts with limited access to bilingual rehabilitation programs. Indeed, the need to improve limited access to quality rehabilitation services for minorities and underserved bilingual populations has been highlighted in prior research (6, 23, 57). Therefore, evidence that telerehabilitation can be implemented with equivalent effectiveness and reliability for BWA as in conventional in-person therapy can (i) promote its use in clinical practice and inclusion in health insurance coverage, (ii) increase awareness regarding the availability of alternative modes of delivering healthcare resources, (iii) motivate positive attitudes toward teletherapy in patients and caregivers with limited technology knowledge and experience, and (iv) facilitate access to bilingual clinical services for BWA with a variety of cultural backgrounds and language combinations. Validated methods of telerehabilitation may ultimately contribute to reducing health disparities for BWA belonging to cultural and linguistic minorities by increasing their opportunities to equal the standard of care available for monolinguals and minimizing the effects of socioeconomic inequities that may influence their limited accessibility to in-person bilingual treatment clinics.

A few limitations of this work should be considered. This is a retrospective study with a small sample including patients who received teletherapy because they could not attend in-person treatment sessions. Also, in accordance with the goal of our ongoing RCT, patients were randomly allocated to a model-prescribed experimental group or a model-opposite control group instead of using a random assignment according to mode of treatment delivery. Because of the reduced sample size and the retrospective approach of our study, these findings should be considered as preliminary evidence supporting the treatment effectiveness and reliability of teletherapy relative to in-person therapy. However, future research employing non-inferiority prospective clinical trials with larger samples and randomized assignment to each delivery method should be conducted to corroborate these findings.

To conclude, our study findings support the effectiveness and reliability of telerehabilitation as a mode of delivering semantic-based therapy for BWA across different individual profiles of bilingualism and impairment, and recommend its use in the context of clinical trials. As technology evolves to accommodate individuals with language and motor deficits and the use of videoconference to deliver therapy becomes more widespread, telerehabilitation may further show increased potential to provide more linguistic and culturally relevant treatments for this population.

## Data Availability Statement

The raw datasupporting the conclusions of this article will be made available upon request to the authors.

## Ethics Statement

The procedures involving human participants were reviewed and approved by Boston University Charles River Campus Institutional Review Board. The patients/participants provided their written informed consent to participate in this study. Written informed consent was obtained from the individual(s) for the publication of any potentially identifiable images or data included in this article.

## Author Contributions

SK conceptualized and designed the study. CP wrote the manuscript and developed the therapy protocols for the study. CP and MS designed all treatment fidelity procedures and conducted the analysis and interpretation of the data. CP, MS, AG, and EC were involved in patient assessment and treatment. NM was in charge of patient randomization and IRB procedures. TG and SS coordinated multisite patient recruitment and data collection. All authors contributed to the article and approved the submitted version.

## Conflict of Interest

SK owns ownership stock and is an advisor for Constant Therapy Health with no scientific overlap with the present work. The remaining authors declare that the research was conducted in the absence of any commercial or financial relationships that could be construed as a potential conflict of interest.

## Footnotes

^1^The number of patients in each group differs across comparisons because treatment reliability was initially planned and completed using a crossed design with *N* = 12 patients; however, four patients were added to the full sample (*N* = 16) over the course of the study to increase statistical power to evaluate treatment effectiveness across delivery modalities.

^2^Patients P1, P2, P4, and P6 received therapy using the GoToMeeting software available online at: https://www.gotomeeting.com/ as this was the videoconference platform initially used for this study. However, as the functions “record session,” “screen sharing,” and “remote control” used in this study are largely comparable between GoToMeeting and Zoom, we only describe the relevant procedures involving the Zoom software.
