# Supplementary material for: Telerehabilitation for Word Retrieval Deficits in Bilinguals With Aphasia: Effectiveness and Reliability as Compared to In-person Language Therapy
Source: Front Neurol. 2021 May 20;12:589330. doi: 10.3389/fneur.2021.589330 (PMC8172788; doi:10.3389/fneur.2021.589330)
Supplement: Supplementary file 1 [file Table_1.DOCX]

Supplementary materials

Treatment fidelity scoring form for independent raters

| Patient ID |  |
| --- | --- |
| Videoconference/In-person |  |
| Clinician |  |
| Treatment language |  |
| Session Number |  |
| Treated item number |  |
| Treated item name |  |

| Treatment step | Criteria | Score | Notes |
| --- | --- | --- | --- |
| Step 1: Naming | Clinician asks patient for the name of the item and gives feedback about correctness | 0 1 |  |
| Step 2A: Feature selection | Clinician correctly identifies any errors and provides appropriate reinforcement | 0 0.5 1 |  |
| Step 2B: Feature assignment | Clinician correctly identifies any errors and provides appropriate reinforcement | 0 0.5 1 |  |
| Step 3: Association | Clinician asks for an association and provides feedback appropriate for the response given | 0 1 |  |
| Step 4: Yes/No questions | Clinician correctly identifies any errors and provides appropriate reinforcement | 0 0.5 1 |  |
| Step 5: Naming | Clinician asks patient for the name of the item and gives feedback about correctness | 0 1 |  |
| Step 6: Sentence production | Clinician elicits sentence and provides feedback about correctness of sentence | 0 1 |  |
